# Supplementary material for: The interactome of intact mitochondria by cross-linking mass spectrometry provides evidence for coexisting respiratory supercomplexes
Source: Mol Cell Proteomics. 2017 Dec 8;17(2):216–32. doi: 10.1074/mcp.RA117.000470 (PMC5795388; doi:10.1074/mcp.RA117.000470)
Supplement: Supplemental Data [file supp_RA117.000470_133922_0_supp_23978_fzffwf.zip › spectra_annotation/mito_DR_spectra_annotation/478-1-2-1-15-1.pdf]

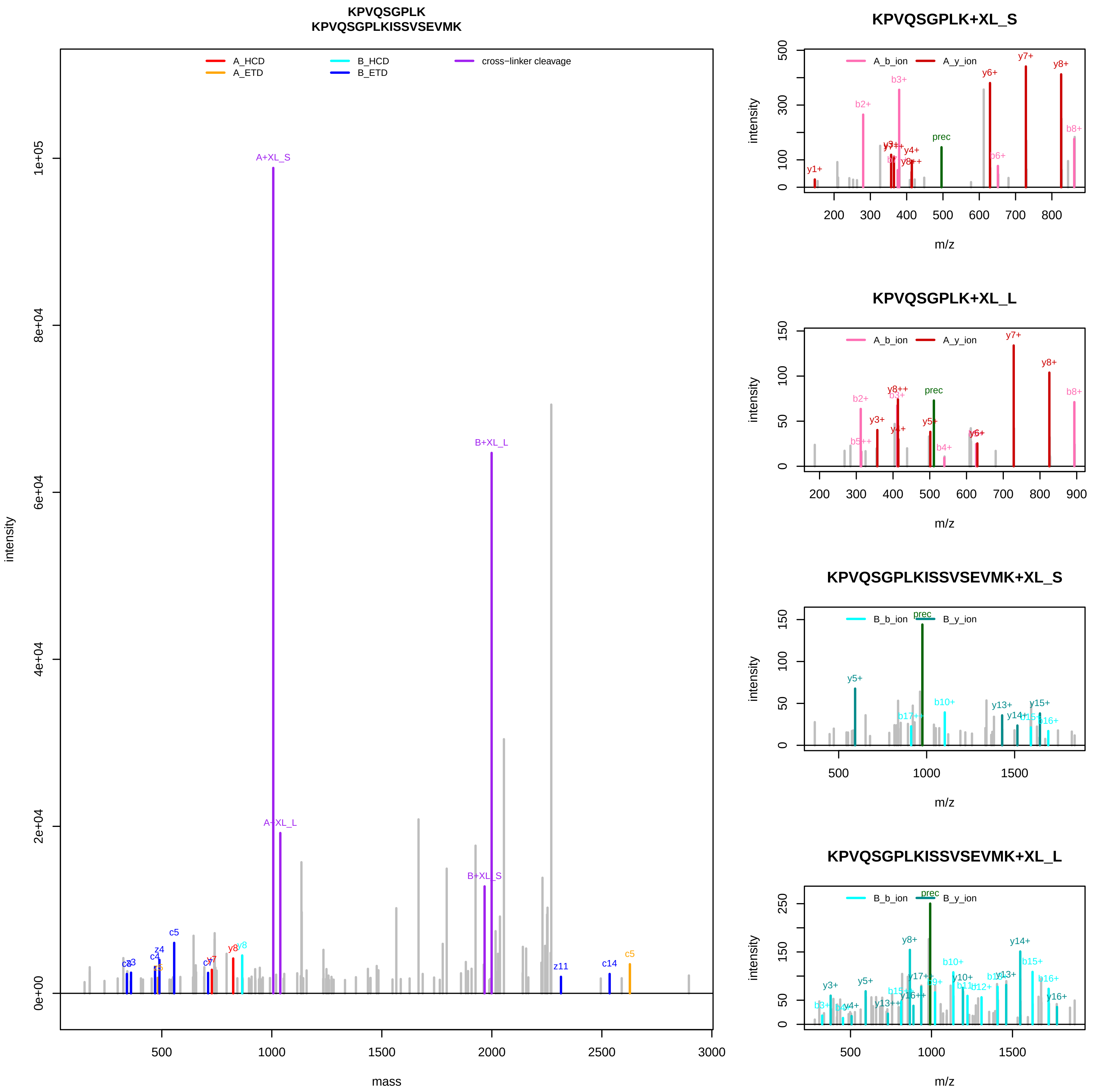
KPVQSGPLK
KPVQSGPLKISSVSEVMK
A_HCD
A_ETD
B_HCD
B_ETD
cross−linker cleavage
A+XL_S
B+XL_L
A+XL_L
B+XL_S
c5
z4
c4
z3
c3
y8
y7
c7
z11
c14
intensity
mass
0e+00
2e+04
4e+04
6e+04
8e+04
1e+05
500
1000
1500
2000
2500
3000
KPVQSGPLK+XL_S
A_b_ion
A_y_ion
y1+
b2+
b3+
y6+
y7+
y8+
b8+
b6+
y4+
prec
intensity
m/z
KPVQSGPLK+XL_L
y3+
y5+
y6+
b4+
b5++
y8++
KPVQSGPLKISSVSEVMK+XL_S
B_b_ion
B_y_ion
y5+
b10+
y13+
y14+
y15+
b16+
KPVQSGPLKISSVSEVMK+XL_L
y3+
y5+
y8+
b10+
y10+
y13+
y14+
b15+
b16+
y16+
